# Region-specific growth restriction of brain following preterm birth

**DOI:** 10.1038/srep33995

**Published:** 2016-09-23

**Authors:** Sachiko Iwata, Reiji Katayama, Masahiro Kinoshita, Mamoru Saikusa, Yuko Araki, Sachio Takashima, Toshi Abe, Osuke Iwata

**Affiliations:** 1 Department of Paediatrics and Child Health, Kurume University School of Medicine, Fukuoka, Japan; 2 Centre for Developmental and Cognitive Neuroscience, Kurume University School of Medicine, Fukuoka, Japan; 3 Diagnostic Imaging Centre, Kurume University Hospital, Fukuoka, Japan; 4 Faculty of Informatics, Shizuoka University, Hamamatsu, Shizuoka, Japan; 5 Yanagawa Institute for Developmental Disabilities, International University of Health and Welfare, Fukuoka, Japan; 6 Department of Radiology, Kurume University School of Medicine, Fukuoka, Japan

## Abstract

Regional brain sizes of very-preterm infants at term-equivalent age differ from those of term-born peers, which have been linked with later cognitive impairments. However, dependence of regional brain volume loss on gestational age has not been studied in detail. To investigate the spatial pattern of brain growth in neonates without destructive brain lesions, head MRI of 189 neonates with a wide range of gestational age (24–42 weeks gestation) was assessed using simple metrics measurements. Dependence of MRI findings on gestational age at birth (Age_birth_) and the corrected age at MRI scan (Age_MRI_) were assessed. The head circumference was positively correlated with Age_MRI_, but not Age_birth_. The bi-parietal width, deep grey matter area and the trans-cerebellar diameter were positively correlated with both Age_birth_ and Age_MRI_. The callosal thickness (positive), atrial width of lateral ventricle (negative) and the inter-hemispheric distance (negative) were exclusively correlated with Age_birth_. The callosal thickness and cerebral/cerebellar transverse diameters showed predominant dependence on Age_birth_ over Age_MRI_, suggesting that brain growth after preterm-birth was considerably restricted or even became negligible compared with that *in utero*. Such growth restriction after preterm birth may extensively affect relatively more matured infants, considering the linear relationships observed between brain sizes and Age_birth_.

During the last decade, magnetic resonance imaging (MRI) scans at term-equivalent age have been established as a reliable prognostic biomarker of motor, verbal and cognitive outcomes in preterm infants^1^,^2^,^3^. In addition to the detection of overt destructive brain injury, evaluation of subtle brain injury, represented by diffuse-excessive high signal intensity and mild brain atrophy, has been established using a composite assessment scale for both white matter and grey matter^2^. For more objective assessment of regional brain sizes, quantitative analysis of brain MRI has been developed. Three-dimensional volumetric analysis gives direct measures of regional brain volume^4^,^5^, where reduced brain volume in preterm infants is indicative of adverse neurodevelopmental outcomes up to 2 years of age^6^,^7^. A more recent study using the same technique demonstrated that low brain volumes observed in very-preterm infants are associated with long-term functional outcomes of up to 7 years old^8^. Even without specialised software and expertise, reliable regional brain sizes can be obtained using simple biometric analysis^9^. Although a relatively greater inter-observer variability was noted for the measurement of fluid spaces^9^, Kidokoro and colleagues reported that the predictive value of cognitive development may be improved by incorporating one-dimensional measurements of regional brain sizes into the aforementioned composite MRI scoring system^10^.

Brain sizes obtained using simple metric analysis in the parietal and frontal lobes, and cerebellum show consistent dependences on the corrected age of preterm infants at the MRI scan (dependence on gestational age at birth not assessed)^9^. This finding suggests that brain growth after preterm birth at least mimics that within the uterus. However, previous comparative studies showed a significant reduction of regional brain volume in very-preterm infants compared with their full-term peers when assessed at term-equivalent age, suggesting a difference between intra- and extra-uterine patterns of brain growth^11^,^12^,^13^.

Important questions are raised whether regional brain volume loss is specific to very-preterm infants or is extensively observed in a gestational-age dependent manner, and whether smaller regional brain sizes at term represent the consequence of permanent brain injury or a temporary delay in growth that can eventually catch up. However, the spatial patterns of altered regional brain growth and their mechanism have not been fully elucidated especially in moderately- and late-preterm infants.

To investigate spatial growth patterns of the brain in preterm infants, we performed an MRI study using simple metrics measurement. Instead of comparing MRI findings between several groups of preterm and term infants, we assessed the dependences of regional brain sizes on the gestational age at birth (Age_birth_) and corrected age at the MRI scan (Age_MRI_) in a single cohort of newborn infants spanning a wide range of gestational ages. This was based on an assumption that, in specific brain regions, where brain growth following preterm birth is substantially restricted, regional brain sizes may depend on Age_birth_ rather than Age_MRI_.

## Results

### Clinical characteristics

Five newborn infants were diagnosed with congenital cerebral anomalies (congenital hydrocephaly, n = 1; major chromosomal abnormality, n = 2; and congenital cytomegalovirus infection, n = 2), and 11 newborn infants showed severe, destructive cerebral lesions (intra-ventricular haemorrhage ≥ grade 3, n = 5; cerebral venous or arterial infarction, n = 3; and cystic encephalomalacia due to severe neonatal encephalopathy, n = 3). Of these 16 newborn infants, moderate to severe brain injury in white matter, cortical grey matter, deep grey matter, cerebellum and the whole brain was observed in 10 (62.5%), 2 (12.5%), 7 (43.8%), 7 (43.8%) and 10 (62.5%) newborn infants, respectively. These subjects were excluded from further analysis.

Subsequently, MRI findings were assessed for 189 preterm and term infants, whose Age_birth_ and Age_MRI_ were 31.8 ± 4.1 (range, 22.6–42.0) weeks and 38.9 ± 1.6 (range, 36.3–44.3) weeks, respectively (Table 1). Age_MRI_ was positively correlated with Age_birth_ (*p* = 0.021). Antenatal and/or postnatal glucocorticoids were used in 43.3% and 15.3% of the population, respectively; 66.1% of the newborn infants were born via caesarean section; mechanical ventilation was required in 49.7%.

### MRI findings

Moderate to severe brain injury in white matter, cortical grey matter, deep grey matter, cerebellum and the whole brain was observed in 15 (7.9%), 1 (0.5%), 0 (0.0%), 8 (4.3%), and 4 (2.1%) newborn infants, respectively (Supplemental Table 1). Moderate to severe injury in the whole brain was exclusively observed in very-preterm infants <28 weeks gestation. Measures from simple metric assessments are presented in Table 2.

### Dependence of MRI findings on gestational and corrected age

**Figure 1:**
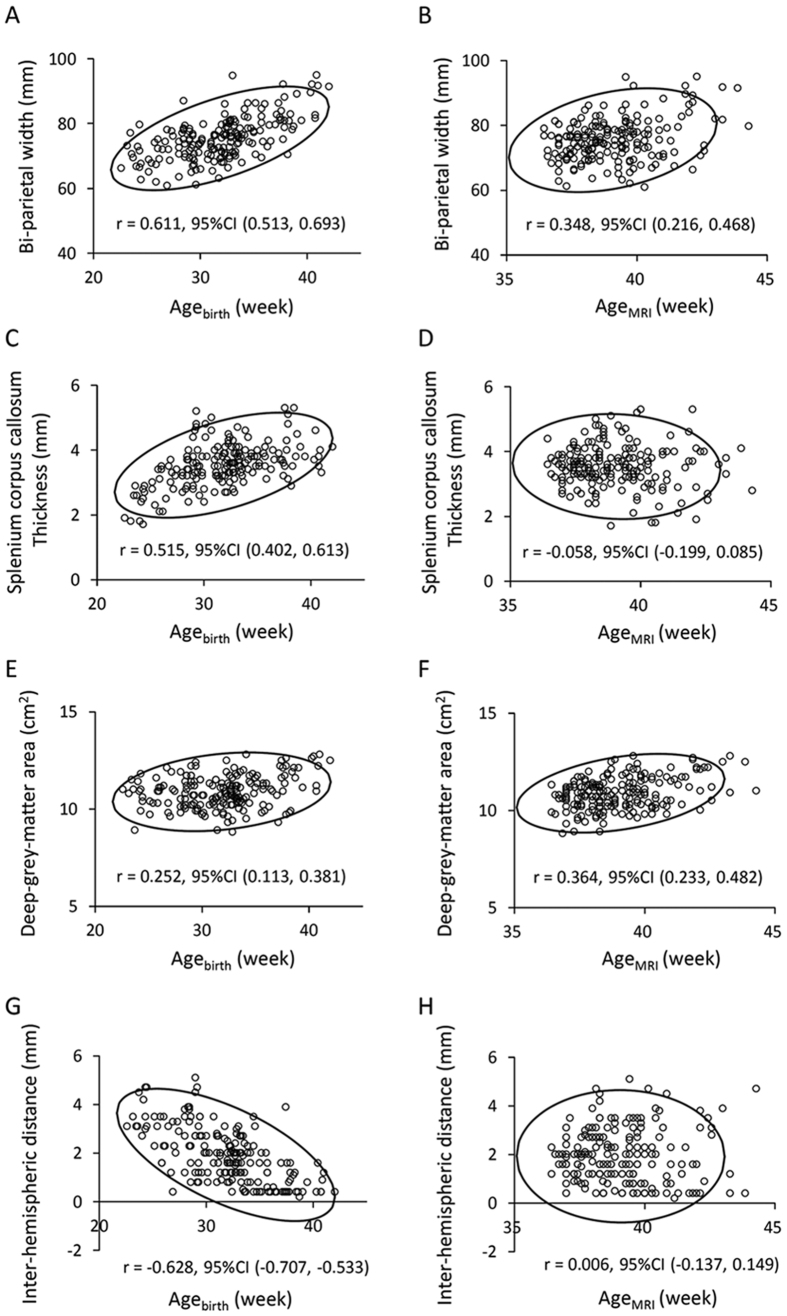
Relationships between regional brain sizes and age. Regional brain sizes are plotted against gestational age at birth (Age_birth_) (**A,C,E,G**) and corrected age at MRI scan (Age_MRI_) (**B,D,F,H**) with 95% confidence ellipse. The bi-parietal width (**A,B**) and deep-grey-matter area (**E,F**) were positively correlated with both Age_birth_ and Age_MRI_. The thickness of the splenium of the corpus callosum and inter-hemispheric distance were correlated with Age_birth_, but not Age_MRI_ (**C,D,G,H**).

Of the qualitative evaluation items within the composite MRI scoring system, the status of myelination was dependent on Age_MRI_ (*p* < 0.001), but not Age_birth_ (Supplemental Table 2). The severity of brain injury for the white matter (*p* < 0.001), cortical grey matter (*p* = 0.002), cerebellum (*p* < 0.001) and the whole brain (*p* < 0.001) was linearly associated with Age_birth_, but not Age_MRI_. The bi-parietal width, deep grey matter area and the trans-cerebellar diameter showed positive linear correlations with both Age_MRI_ and Age_birth_ (all *p* < 0.001), which were most prominent between the bi-parietal width and Age_birth_ (Table 2, Fig. 1 and Supplemental Fig. 1; see Supplemental Table 3 for findings from exploratory analysis, which assessed the dependence of regional brain sizes on other clinical variables). The callosal thickness for all three regions (positive) (all *p* < 0.001), atrial width of lateral ventricle (negative) (*p* = 0.001) and the inter-hemispheric distance (negative) (*p* < 0.001) were linearly correlated with Age_birth_, but not with Age_MRI_. The head circumference was positively correlated with Age_MRI_ (*p* < 0.001), but not with Age_birth_.

### Relationship between head circumference and brain metrics

The bi-parietal width was not correlated with the fronto-occipital diameter, whereas a positive relationship was observed between the trans-cerebellar diameter and the antero-posterior cerebellar diameter (*p* < 0.001) (Table 3). The fronto-occipital diameter and the bi-parietal width were linearly correlated with the antero-posterior cerebellar diameter and the trans-cerebellar diameter, respectively (both *p* < 0.001). The deep-grey-matter area was correlated with the bi-parietal width, fronto-occipital diameter, trans-cerebellar diameter, antero-posterior cerebellar diameter (all p < 0.001) and the splenial thickness of the corpus callosum (*p* = 0.001). The atrial width and the thalamo-occipital distance of the lateral ventricle were positively correlated with the fronto-occipital diameter (both *p* < 0.001). The atrial width of the lateral ventricle was correlated with the thalamo-occipital distance of the lateral ventricle (*p* < 0.001), but not with the inter-hemispheric distance. The head circumference was correlated with the bi-parietal width, fronto-occipital diameter, deep-grey-matter area, trans-cerebellar diameter, antero-posterior cerebellar diameter and the thalamo-occipital distance of the lateral ventricle (all *p* < 0.001), where the most robust relationship was seen with the fronto-occipital diameter.

## Discussion

Previous MRI studies have highlighted the poor regional brain growth of preterm infants compared with their term-born peers^9^,^11^,^12^. In the current study, instead of comparing brain sizes between preterm- and term-born cohorts, we assessed the dependence of brain size on Age_birth_ and Age_MRI_ in a single cohort of newborn infants with a spectrum of maturation stages. This was based on an assumption that, in regions where postnatal brain growth mimics that *in utero*, regional brain sizes primarily depend on Age_MRI_, but not Age_birth_. However, we found that measures such as deep-grey-matter area and transverse diameters of the cerebrum and cerebellum depended on both Age_birth_ and Age_MRI_, whereas the thickness of the corpus callosum depended exclusively on Age_birth_. This suggests that brain growth in these regions after preterm-birth is significantly restricted or is even negligible compared with that *in utero*. Considering that abnormal size of the corpus callosum and lateral ventricles are good indicators of cognitive impairment in children and adolescents born prematurely^14^,^15^,^16^,^17^,^18^, the region-specific growth patterns of the immature brain observed in our study may represent the irreversible consequence of adverse extra-uterine conditions for the brain of preterm infants. In addition, given the linear relationships observed between brain sizes and Age_birth_ in some regions, growth restriction of the brain is a continuum, which is not specific to extremely- and very-preterm infants, but may affect even more matured newborn infants.

Despite the established relationship between callosal size and neurodevelopmental outcomes following preterm birth^14^,^15^,^16^,^17^,^18^, the mechanism of altered callosal growth remain largely unknown. Volume loss in the corpus callosum of preterm-born children is most evident in the posterior region^14^,^15^,^17^,^19^. The rudimentary corpus callosum originates in the anterior body at the end of the first trimester, and expands towards remaining parts during development^20^. Similarly, the genu and the body show a consistent increase in thickness during pregnancy^21^,^22^, whereas the splenium shows the maximum growth in thickness between 18 and 26 weeks gestation, which slows down after 28 weeks gestation^22^. Preterm infants, even with their favourable clinical course, experience dramatic environmental changes at birth, which are followed by a prolonged period with stressful procedures, malnutrition, and separation from the mother. The burden of such adverse events, experienced just when the splenium is supposed to grow most rapidly, might be responsible for altered callosal growth after preterm birth.

In preterm infants, increased cerebrospinal fluid space is persistently observed even in adolescence^17^,^23^. Ventriculomegaly without preceding severe intra-ventricular haemorrhage is known as an independent predictive marker for adverse neurodevelopmental outcomes in preterm infants^18^,^24^,^25^. Interestingly, the thalamo-occipital distance of the lateral ventricle measured shortly after birth showed little or no dependence on gestational age^26^,^27^, suggesting that intra-uterine brain growth is accompanied by the reduction of the ventricular size relative to the whole brain. In the current study, the sizes of ventricular and subarachnoid spaces were negatively correlated with Age_birth_, indicating that the growth rate of brain tissue surrounding the ventricle is significantly restricted after preterm birth. In addition, consistent with previous studies^24^,^28^, we observed no direct relationship between the sizes of ventricular and subarachnoid spaces. The primary cause of the increase in cerebrospinal fluid-space volume likely differs between newborn infants, because increase in ventricular and subarachnoid spaces can be caused by the volume reduction of the adjacent brain tissue as well as the increase in cerebrospinal fluid itself.

In the current study, the bi-parietal width and the fronto-occipital diameter were not correlated with each other. Unlike the bi-parietal width, the fronto-occipital diameter showed no correlation with Age_birth_ and Age_MRI_. However, the fronto-occipital diameter was tightly correlated with both the head circumference and the thalamo-occipital distance of the lateral ventricle, suggesting that the brain size along the longitudinal axis might be determined by both physiological postnatal growth and pathological dilatation of ventricles. This characteristic balance of cerebral growth between the longitudinal and transverse axes may explain the scaphocephalic head shape commonly observed in preterm infants. It is widely accepted that scaphocephaly in healthy preterm infants does not influence deep brain structures^29^. However, the current findings suggest that excessive scaphocephaly might be indicative of altered brain volume and structure subsequent to preceding brain injury. In addition, given that approximately 51% of the head circumference can be explained by the fronto-occipital diameter (based on the observed r^2^ = 0.51 between these variables), careful interpretation is required to assess the brain growth using the head circumference in preterm infants.

Although no correlation was observed between the bi-parietal width and the fronto-occipital diameter, the longitudinal and transverse diameters of the cerebrum were tightly correlated with those of the cerebellum. The transverse diameters of the cerebrum and the cerebellum both showed predominant correlations with Age_birth_ despite significant differences in the environment between the supra- and infra-tentorial spaces. This suggests the presence of a common mechanism causing the growth restriction of the cerebrum and cerebellum along the transverse axis in preterm infants. Considering that the sideways head position is generally preferred for preterm infants during intensive care, gravity may predominantly affect brain growth towards the transverse axis of the brain after birth. This may influence the growth pattern of the immature brain, as studies in developing rat demonstrated that exposure to hyper-gravity environment causes various types of cerebellar injury, including Purkinje cell loss and subsequent reduction in cerebellar volume^30^.

In the current study, the severity of brain injury assessed using the composite MRI scores depended on Age_birth_, where Age_MRI_ is already incorporated within the scoring system^10^. Given that we did not include newborn infants with major cerebral lesions in the current cohort, preterm birth itself was likely to be the primary independent variable of non-destructive brain injury at term. Recently, the incidence of moderate to severe neurodevelopmental impairments in preterm infants has decreased, in part due to the reduced incidence of destructive brain lesions such as intra-ventricular haemorrhage and periventricular leukomalacia^31^,^32^. Hence, early diagnosis of non-destructive brain lesions would be important for the prevention of subsequent cognitive impairment. Comprehensive assessment of brain growth and maturation at term using qualitative measures, regional brain size and other quantitative markers may allow more precise detection of injury.

We excluded newborn infants with apparent destructive brain lesions. This led to uncertainty regarding typical brain growth in extremely-preterm infants, who often develop severe cerebral lesions. We did not use volumetric analysis in our current study. While volumetric data are easy to translate, their use in clinical practice remains limited because of additional requirements for software and expertise for data processing. The present study and others suggest the benefit of a simple metric approach, which is reliable, reproducible and readily available in clinical practice^9^. Future studies should incorporate other quantitative magnetic resonance biomarkers including apparent diffusion coefficients, fractional anisotropy and T2-relaxation time.

We found that the size of specific brain regions, including the thickness of the corpus callosum and the transverse diameter of the cerebrum, depended on Age_birth_, but not Age_MRI_, suggesting a difference between intra- and extra-uterine brain growth after preterm birth. The linear relationships observed between brain sizes and Age_birth_ in these regions suggested that regional brain growth restriction is not specific to very-preterm infants, but is likely to affect even more matured infants in a gestational-age dependent manner. Further studies are required to elucidate the mechanism and direct consequence of brain growth patterns specific to preterm infants. Serial cranial ultrasound sonography might help delineate the temporal process of region-specific growth pattern in these infants.

## Methods

This study was conducted in compliance with the Declaration of Helsinki under the approval of the Ethics Committee of Kurume University School of Medicine. Informed parental consent was obtained for each participating newborn infant before enrolment into this study.

### Study population

Two hundred and five newborn infants, who were admitted to a tertiary neonatal intensive care unit of Kurume University Hospital (Kurume, Fukuoka, Japan) immediately after birth and underwent MRI scans between 36 and 44 weeks corrected age during the period from September 2007 to March 2012, were enrolled into the study. In this unit, head MRI is routinely obtained (i) for preterm infants <34 weeks gestation, and (ii) for near term- and term-born infants with either neurological abnormality, clinical details suggestive of perinatal hypoxia–ischaemia, respiratory failure, major congenital anomaly, chromosome aberration, or metabolic disease, after the clinical condition is stabilised.

### MRI study

**Figure 2:**
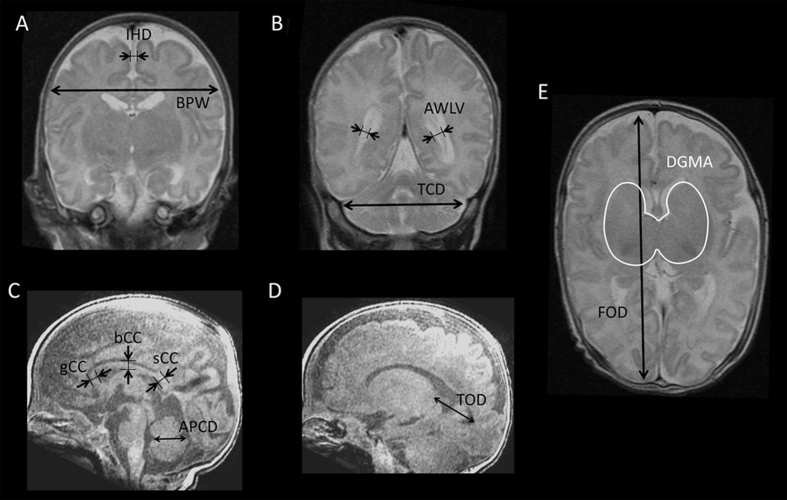
Representative images showing the measurement of regional brain sizes. A coronal section at the level of the cochlea and basilar truncus apparent (**A**) and the lateral ventricular atrium (**B**) are measured for the bi-parietal width (BPW) and interhemispheric distance (IHD) on A and for the atrial width of each lateral ventricle (AWLV) and trans-cerebellar diameter (TCD) on B. A midline sagittal section (**C**) was used for the measurement of callosal thickness at the genu (gCC), mid-portion of the body (bCC), and splenium (sCC), and antero-posterior cerebellar diameter (APCD). A sagittal section, with which a plain from the thalamus to the occipital horn is maximally visible (**D**), was used for the measurement of the thalamo-occipital distance of the lateral ventricle (TOD). An axial section, with which the caudate, lentiform nuclei and the thalamus are observed with their maximal sizes (**E**), was used for the measurement of the fronto-occipital diameter (FOD) and the deep-grey-matter area (DGMA).

A 3-Tesla Signa HDxt scanner (GE Medical Systems, Milwaukee, WI, USA) was used to obtain head MRI with a three-dimensional brain volume imaging (BRAVO) for T1-weighted images (TR 11 ms; TE 5 ms; slice thickness 1 mm; matrix 384 × 224, interpolated 512 × 512; field of view 200 × 200 mm; both coronal and sagittal sections reconstructed from axial slices) and a fast spin echo imaging for T2-weighted images (TR 5000 ms; TE 87 ms; slice thickness 4 mm; matrix 384 × 224, reconstruction matrix 512 × 512; field of view 200 × 200 mm). Diffusion-tensor imaging was also obtained, information of which was not used in the current study. MRI was visually inspected for its quality, and was assessed using an established MRI scoring system for brain maturation, growth, and injury^10^. This system gives 0–4 stepwise scores over 13 items, according to the qualitative and quantitative findings of the brain on T1- and T2-weighted images, so that composite scores are calculated for the white matter (range, 0–17), cortical grey matter (0–9), deep grey matter (0–7) and cerebellum (0–7). The MRI was categorised as having no, mild, moderate, or severe injury according to the regional and global composite scores (Supplemental Fig. 2). Simple metric measures were obtained for T2-weighted coronal images (bi-parietal width, trans-cerebellar diameter, interhemispheric distance, and atrial width of the lateral ventricle), T1-weighted sagittal images (thalamo-occipital distance of the lateral ventricle, thickness of the corpus callosum at the genu, mid-portion of the body, and splenium, and antero-posterior cerebellar diameter) and T2-weighted axial images (fronto-occipital diameter and deep-grey-matter area) sections^9^ (Fig. 2).

### Clinical information

Clinical information for the newborn infants was obtained including intrauterine growth restriction, gestational age and body weight at birth, delivery mode, use of antenatal and postnatal glucocorticoid, duration of mechanical ventilation, postnatal age when enteral feeding exceeded 100 mL/kg, symptomatic patent ductus arteriosus requiring treatments with intravenous indomethacin (excluding prophylactic administration within 72 h of life) or surgical ligation, chronic lung disease (oxygen dependence on Day 28 and/or 36 weeks corrected age), and the body weight and head circumference on the day of the MRI scan.

### Data analysis

To further understand the extra-uterine brain growth of newborn infants without major anomalies and destructive brain injury, newborn infants with congenital cerebral anomalies or destructive brain lesions (intra-ventricular haemorrhage ≥ grade 3, cerebral infarction and cystic encephalomalacia due to severe neonatal encephalopathy) were excluded from the analysis. Correlations between brain metrics, head circumference on the day of the MRI scan, severity of MRI findings on composite scores, Age_birth_ and Age_MRI_ were assessed using either Pearson’s correlation coefficient or Spearman’s correlation coefficient when applicable. For multiple comparisons over 11 simple brain metrics and 18 items of the composite scoring system, statistical significance was assumed for *p* < 0.0045 and 0.0028, respectively (Bonferroni correction).

## Additional Information

**How to cite this article**: Iwata, S. *et al*. Region-specific growth restriction of brain following preterm birth. *Sci. Rep.* **6**, 33995; doi: 10.1038/srep33995 (2016).

## Supplementary Material

Supplementary Information

## Figures and Tables

**Table 1:** Clinical characteristics of the study population.

Variables	Mean ± SD, or number (%)
Gestational age at birth (week)	31.8 ± 4.1
<28	29 (15.3)
28 ≤<32	58 (30.7)
32 ≤<36	73 (38.6)
37≤	29 (15.3)
Birth weight (g)	1537 ± 709
Male sex	91 (48.1)
Antenatal glucocorticoid	82 (43.3)
Multiple pregnancy	50 (26.5)
Caesarean delivery	125 (66.1)
Intrauterine growth restriction	71 (37.6)
Apgar score <7	
1 min.	90 (47.6)
5 min.	29 (15.3)
Duration of mechanical ventilation (day)	9.8 ± 17.5
Chronic lung disease	45 (23.8)
Symptomatic patent ductus arteriosus	
Indomethacin	49 (25.9)
ligation	10 (5.3)
Enteral feeding >100 ml/kg (day)	7.6 ± 5.5
Postnatal glucocorticoid	29 (15.3)
Corrected age at MRI scan (week)	38.9 ± 1.6
Head circumference at MRI scan (cm)	34.2 ± 1.6

Abbreviation: SD, standard deviation.

**Table 2:** Relationships between simple brain metrics and age.

Variables	Mean	SD	Age_birth_			Age_MRI_		
			r	(95% CI)	p	r	(95% CI)	p
Head circumference (cm)	34.2	1.6	0.120	(−0.023, 0.258)	0.100	0.447	(0.325, 0.554)	<0.001
Cerebral hemisphere								
Bi-parietal width (mm)	75.4	6.5	0.611	(0.513, 0.693)	<0.001	0.348	(0.216, 0.468)	<0.001
Fronto-occipital diameter (mm)	100.3	5.3	−0.004	(−0.147, 0.147)	0.957	0.067	(−0.076, 0.208)	0.358
Corpus callosum (thickness in [mm])								
Genu	4.4	0.8	0.332	(0.199, 0.453)	<0.001	0.139	(−0.004, 0.276)	0.056
Body	2.5	0.5	0.328	(0.194, 0.450)	<0.001	0.078	(−0.065, 0.218)	0.287
Splenium	3.5	0.7	0.515	(0.402, 0.613)	<0.001	−0.058	(−0.199, 0.085)	0.426
Deep grey matter								
Deep-grey-matter area (cm^2^)	10.9	0.8	0.252	(0.113, 0.381)	<0.001	0.364	(0.233, 0.482)	<0.001
Cerebellum								
Trans-cerebellar diameter (mm)	50.2	2.8	0.561	(0.455, 0.652)	<0.001	0.483	(0.365, 0.585)	<0.001
Antero-posterior cerebellar diameter (mm)	16.4	1.4	0.105	(−0.038, 0.244)	0.151	0.367	(0.237, 0.484)	<0.001
Fluid measures								
Atrial width of Lateral ventricle (mm)								
Right	5.1	1.6						
Left	6.4	2.1						
Mean	5.8	1.7	−0.232	(−0.363, −0.092)	0.001	0.061	(−0.082, 0.202)	0.404
Thalamo-occipital distance (mm)	25.5	4.3	−0.165	(−0.301, −0.023)	0.024	−0.043	(−0.185, 0.100)	0.552
Inter-hemispheric distance (mm)	1.9	1.1	−0.628	(−0.707, −0.533)	<0.001	0.006	(−0.137, 0.149)	0.936

Abbreviations: SD, standard deviation; Age_birth_, gestational age at birth; Age_MRI_, corrected age at MRI scan. P-values are from the Pearson’s correlation coefficient, using the Fisher Transformation to calculate the 95% confidence intervals.

**Table 3:** Correlation coefficient and 95% confidence interval between regional brain sizes.

	BPW	FOD	gCC	bCC	sCC	DGMA	TCD	APCD	AWLV	TOD	IHD
Head circumference (HC)	0.418	0.713	0.025	0.154	0.279	0.574	0.585	0.560	0.183	0.397	0.029
	(0.293, 0.529)	(0.635, 0.777)	(−0.118, 0.167)	(0.012, 0.290)	(0.142, 0.406)	(0.470, 0.662)	(0.483, 0.672)	(0.454, 0.651)	(0.041, 0.317)	(0.270, 0.511)	(−0.114, 0.171)
Bi-parietal width (BPW)		−0.043	0.176	0.201	0.336	0.355	0.674	0.193	−0.231	−0.173	−0.207
		(−0.185, 0.100)	(0.034, 0.311)	(0.060, 0.334)	(0.203, 0.457)	(0.224, 0.474)	(0.588, 0.745)	(0.052, 0.327)	(−0.362, −0.091)	(−0.308, −0.031)	(−0.340, −0.066)
Fronto-occipital diameter (FOD)			−0.001	0.061	0.190	0.338	0.246	0.479	0.294	0.480	0.049
			(−0.144, 0.142)	(−0.082, 0.202)	(0.049, 0.324)	(0.205, 0.459)	(0.107, 0.376)	(0.361, 0.582)	(0.158, 0.419)	(0.362, 0.583)	(−0.094, 0.190)
Genu of the corpus callosum (gCC)				0.448	0.329	0.172	0.232	0.018	−0.049	−0.010	−0.174
				(0.326, 0.555)	(0.195, 0.451)	(0.030, 0.307)	(0.092, 0.363)	(−0.125, 0.160)	(−0.190, 0.094)	(−0.153, 0.133)	(−0.309, −0.032)
Body of the corpus callosum (bCC)					0.453	0.173	0.253	0.084	−0.065	−0.043	−0.272
					(0.332, 0.560)	(0.031, 0.308)	(0.114, 0.382)	(−0.059, 0.224)	(−0.206, 0.078)	(−0.185, 0.100)	(−0.399, -.134)
Splenium of the corpus callosum (sCC)						0.255	0.363	0.153	−0.084	0.057	−0.384
						(0.117, 0.384)	(0.232, 0.481)	(0.010, 0.289)	(−0.224, 0.059)	(−0.086, 0.198)	(−0.499, -.255)
Deep-grey-matter area (DGMA)							0.506	0.352	0.178	0.237	−0.048
							(0.392, 0.605)	(0.220, 0.471)	(0.036, 0.313)	(0.098, 0.367)	(−0.189, 0.095)
Trans-cerebellar diameter (TCD)								0.356	−0.034	0.064	−0.164
								(0.225, 0.475)	(−0.176, 0.109)	(−0.079, 0.205)	(−0.300, −0.022)
Antero-posterior cerebellar diameter (APCD)									0.065	0.143	0.011
									(−0.078, 0.206)	(0.000, 0.280)	(−0.132, 0.153)
Atrial width of Lateral ventricle (AWLV)										0.533	0.145
										(0.422, 0.628)	(0.002, 0.282)
Thalamo-occipital distance (TOD)											0.103
											(−0.040, 0.242)

Associations between regional brain sizes were assessed with Pearson correlation, using the Fisher Transformation to calculate the 95% confidence intervals. Abbreviation, IHD, inter-hemispheric distance.
