# Supplementary material for: Region-specific growth restriction of brain following preterm birth
Source: Sci Rep. 2016 Sep 23;6:33995. doi: 10.1038/srep33995 (PMC5034268; doi:10.1038/srep33995)
Supplement: Supplementary Information [file srep33995-s1.doc]

**Online Supplemental Tables and Figures**

**Title: Region-specific growth restriction of brain following preterm birth**

**Authors:**

Sachiko Iwata, MD, PhD^1,2^, Reiji Katayama, RT^3^, Masahiro Kinoshita, MD^1^, Mamoru Saikusa, MD^1^, Yuko Araki, PhD^4^, Sachio Takashima, MD, PhD^5^, Toshi Abe, MD, PhD^6^, Osuke Iwata, MD, PhD^1,2^

**Affiliations:**

1. Department of Paediatrics and Child Health, Kurume University School of Medicine, Fukuoka, Japan

2. Centre for Developmental and Cognitive Neuroscience, Kurume University School of Medicine, Fukuoka, Japan

3. Diagnostic Imaging Centre, Kurume University Hospital, Fukuoka, Japan

4. Faculty of Informatics, Shizuoka University, Hamamatsu, Shizuoka, Japan

5. Yanagawa Institute for Developmental Disabilities, International University of Health and Welfare, Fukuoka, Japan

6. Department of Radiology, Kurume University School of Medicine, Fukuoka, Japan

Online Supplemental Table 1:

Prevalence of brain injury assessed using the MRI composite scoring system

Online Supplemental Table 2:

Correlations between MRI composite scores and age

Online Supplemental table 3:

Relationships between simple brain metrics and clinical variables

Online Supplemental Fig. 1:

Relationships between regional brain sizes and age

Online Supplemental Fig. 2:

Representative MRI of newborn infants in the study cohort

**Online Supplemental Table 1: Prevalence of brain injury assessed using the MRI composite scoring system**

**A: Findings for all regions and items**

| Regions and items | | Score 0 | Score 1 | Score 2 | Score 3 | Score 4 |
| --- | --- | --- | --- | --- | --- | --- |
| White matter | |  |  |  |  |  |
|  | Cystic lesion | 177 (93.7) | 2 (1.1) | 10 (5.3) | 0 | 0 |
|  | Focal signal abnormality | 177 (93.7) | 5 (2.6) | 6 (3.2) | 1 (0.5) | 0 |
|  | Myelination delay | 25 (13.2) | 141 (74.6) | 23 (12.2) |  |  |
|  | Thinning of the corpus callosum | 182 (96.3) | 7 (3.7) | 0 |  |  |
|  | Dilated lateral ventricles | 147 (77.8) | 26 (13.8) | 13 (6.9) | 3 (1.6) |  |
|  | Volume reduction | 95 (50.3) | 59 (31.2) | 20 (10.6) | 15 (7.9) |  |
| Cortical grey matter | |  |  |  |  |  |
|  | Signal abnormality | 189 (100) | 0 | 0 | 0 | 0 |
|  | Gyral maturation | 187 (98.9) | 2 (1.1) | 0 |  |  |
|  | Increased extracerebral space | 182 (96.3) | 6 (3.2) | 1 (0.5) | 0 |  |
| Deep grey matter | |  |  |  |  |  |
|  | Signal abnormality | 189 (100) | 0 | 0 | 0 | 0 |
|  | Volume reduction | 186 (98.4) | 3 (1.6) | 0 | 0 |  |
| Cerebellum | |  |  |  |  |  |
|  | Signal abnormality | 185 (97.9) | 1 (0.5) | 2 (1.1) | 1 (0.5) |  |
|  | Volume reduction | 130 (68.8) | 51 (27.0) | 6 (3.2) | 2 (1.1) |  |

**B: Findings for regional and global brain**

| Abnormality grade | | No | Mild | Moderate | Severe |
| --- | --- | --- | --- | --- | --- |
| Regional brain score | |  |  |  |  |
|  | White matter | 114 (60.3) | 60 (31.7) | 11 (5.8) | 4 (2.1) |
|  | Cortical grey matter | 180 (95.2) | 8 (4.2) | 1 (0.5) | 0 |
|  | Deep grey matter | 186 (98.4) | 3 (1.6) | 0 | 0 |
|  | Cerebellum | 129 (68.3) | 52 (27.5) | 6 (3.2) | 2 (1.1) |
| Global brain score | | 132 (69.8) | 53 (28.0) | 4 (2.1) | 0 |

Data are shown as number (percent).

**Online Supplemental Table 2: Correlations between MRI composite scores and age**

| MRI composite scoring | | Age_birth_ | | |  | Age_MRI_ | | |
| --- | --- | --- | --- | --- | --- | --- | --- | --- |
|  |  | r | (95%CI) | p |  | r | (95%CI) | p |
| White matter | |  |  |  |  |  |  |  |
|  | Cystic lesion | -0.010 | (-0.153, 0.133) | 0.887 |  | 0.019 | (-0.124, 0.161) | 0.794 |
|  | Focal signal abnormality | 0.078 | (-0.065, 0.218) | 0.288 |  | -0.066 | (-0.207, 0.077) | 0.364 |
|  | Myelination delay | -0.095 | (-0.235, 0.048) | 0.195 |  | -0.589 | (-0.675, -0.487) | <0.001 |
|  | Thinning of the corpus callosum | -0.296 | (-0.421, -0.160) | <0.001 |  | 0.143 | (0.000, 0.280) | 0.049 |
|  | Dilated lateral ventricles | -0.130 | (-0.268, 0.013) | 0.075 |  | 0.020 | (-0.123, 0.162) | 0.789 |
|  | Volume reduction | -0.504 | (-0.603, -0.389) | <0.001 |  | 0.094 | (-0.049, 0.234) | 0.199 |
| Abnormality grade | | -0.362 | (-0.480, -0.231) | <0.001 |  | -0.044 | (-0.186, 0.099) | 0.549 |
|  | |  |  |  |  |  |  |  |
| Cortical grey matter | |  |  |  |  |  |  |  |
|  | Signal abnormality | NA | | |  | NA | | |
|  | Gyral maturation | -0.004 | (-0.147, 0.139) | 0.959 |  | 0.146 | (0.003, 0.283) | 0.046 |
|  | Increased extra-cerebral space | -0.252 | (-0.381, -0.113) | <0.001 |  | 0.090 | (-0.053, 0.230) | 0.220 |
| Abnormality grade | | -0.226 | (-0.357, -0.086) | 0.002 |  | 0.149 | (0.006, 0.286) | 0.041 |
|  | |  |  |  |  |  |  |  |
| Deep grey matter | |  |  |  |  |  |  |  |
|  | Signal abnormality | NA | | |  | NA | | |
|  | Volume reduction | -0.063 | (-0.204, 0.080) | 0.387 |  | -0.135 | (-0.272, 0.008) | 0.064 |
| Abnormality grade | | -0.063 | (-0.204, 0.080) | 0.387 |  | -0.135 | (-0.272, 0.008) | 0.064 |
|  | |  |  |  |  |  |  |  |
| Cerebellum | |  |  |  |  |  |  |  |
|  | Signal abnormality | 0.247 | (0.108, 0,376) | 0.001 |  | 0.233 | (0.093, 0.364) | 0.001 |
|  | Volume reduction | -0.440 | (-0.548, -0,317) | <0.001 |  | 0.121 | (-0.022, 0.259) | 0.098 |
| Abnormality grade | | -0.420 | (-0.531, -0.295) | <0.001 |  | 0.139 | (-0.004, 0.276) | 0.057 |
|  | |  |  |  |  |  |  |  |
| Global brain abnormality grade | | -0.441 | (-0.549, -0.318) | <0.001 |  | 0.016 | (-0.127, 0.158) | 0.831 |
|  |  |  |  |  |  |  |  |  |

Abbreviations: NA, not applicable due to the lack of incidence; Age_birth_, gestational age at birth; Age_MRI_, corrected age at MRI scan.

P-values are from the Spearman’s correlation coefficient.

**Online Supplemental Table 3: Relationships between simple brain metrics and clinical variables**

| Variables |  | | Body weight | | Female sex | Glucocorticoid | | Multiple birth | C/S | Low Apgar | IUGR | PDA ligation | CLD | Delayed feeding |
| --- | --- | --- | --- | --- | --- | --- | --- | --- | --- | --- | --- | --- | --- | --- |
|  |  | | at birth | at MRI |  | maternal | neonatal |  |  |  |  |  |  |  |
| Head circumference | r | | 0.26 | 0.535 | -0.324 | 0.006 | -0.119 | 0.026 | 0.099 | 0.011 | -0.306 | 0.023 | -0.05 | -0.102 |
|  | p | | <0.001 | <0.001 | <0.001 | 0.939 | 0.102 | 0.72 | 0.177 | 0.879 | <0.001 | 0.753 | 0.495 | 0.185 |
|  | | | | |  |  |  |  |  |  |  |  |  |  |
| Cerebral diameters | | | | |  |  |  |  |  |  |  |  |  |  |
| Bi-parietal width | r | | 0.576 | 0.173 | -0.068 | -0.277 | -0.265 | 0.003 | -0.004 | -0.08 | 0.115 | -0.167 | -0.34 | -0.343 |
|  | p | | <0.001 | 0.018 | 0.353 | <0.001 | <0.001 | 0.968 | 0.956 | 0.275 | 0.115 | 0.023 | <0.001 | <0.001 |
| Fronto-occipital  diameter | r | | 0.048 | 0.243 | -0.234 | 0.087 | -0.161 | 0.089 | 0.206 | -0.174 | -0.11 | 0.058 | 0.001 | 0.058 |
|  | p | | 0.508 | 0.001 | 0.001 | 0.235 | 0.027 | 0.221 | 0.005 | 0.017 | 0.131 | 0.435 | 0.991 | 0.45 |
|  | | | | |  |  |  |  |  |  |  |  |  |  |
| Thickness of the corpus callosum | | | | |  |  |  |  |  |  |  |  |  |  |
| Genu | r | | 0.252 | 0.039 | 0.084 | -0.094 | -0.234 | 0.004 | 0.033 | 0.014 | 0.122 | -0.1 | -0.27 | -0.195 |
|  | p | | <0.001 | 0.6 | 0.25 | 0.196 | 0.001 | 0.957 | 0.657 | 0.85 | 0.094 | 0.175 | <0.001 | 0.011 |
| Body | r | | 0.254 | 0.038 | -0.028 | -0.081 | -0.292 | 0.018 | 0.06 | -0.068 | 0.092 | -0.203 | -0.258 | -0.116 |
|  | p | | <0.001 | 0.607 | 0.705 | 0.271 | <0.001 | 0.811 | 0.409 | 0.35 | 0.21 | 0.006 | <0.001 | 0.133 |
| Splenium | r | | 0.434 | 0.003 | -0.024 | -0.145 | -0.449 | 0.05 | 0.079 | -0.198 | -0.026 | -0.24 | -0.451 | -0.287 |
|  | p | | <0.001 | 0.967 | 0.743 | 0.047 | <0.001 | 0.494 | 0.279 | 0.006 | 0.72 | 0.001 | <0.001 | <0.001 |
|  |  | |  |  |  |  |  |  |  |  |  |  |  |  |
| Deep grey matter | | | |  |  |  |  |  |  |  |  |  |  |  |
| Deep-grey-  matter area | r | | 0.293 | 0.293 | -0.288 | -0.055 | -0.124 | -0.021 | 0.026 | 0.022 | -0.083 | 0.135 | -0.067 | -0.064 |
|  | p | | <0.001 | <0.001 | <0.001 | 0.45 | 0.09 | 0.779 | 0.724 | 0.769 | 0.254 | 0.068 | 0.363 | 0.404 |
|  | | | |  |  |  |  |  |  |  |  |  |  |  |
| Cerebellar diameters | | | |  |  |  |  |  |  |  |  |  |  |  |
| Trans-cerebellar | r | 0.565 | | 0.236 | -0.121 | -0.236 | -0.268 | -0.076 | -0.028 | -0.09 | -0.02 | -0.074 | -0.35 | -0.24 |
|  | p | <0.001 | | 0.001 | 0.097 | 0.001 | <0.001 | 0.297 | 0.705 | 0.22 | 0.789 | 0.315 | <0.001 | 0.002 |
| Antero-posterior | r | 0.101 | | 0.228 | -0.189 | -0.06 | -0.039 | -0.092 | 0.03 | -0.047 | 0.059 | 0.022 | 0.052 | 0.061 |
|  | p | 0.167 | | 0.002 | 0.009 | 0.41 | 0.59 | 0.208 | 0.681 | 0.522 | 0.418 | 0.769 | 0.474 | 0.427 |
|  | | | |  |  |  |  |  |  |  |  |  |  |  |
| Fluid measures | | | |  |  |  |  |  |  |  |  |  |  |  |
| Mean atrial width | r | -0.126 | | 0.132 | -0.056 | 0.124 | 0.156 | -0.01 | -0.044 | 0 | -0.129 | 0.018 | 0.149 | 0.12 |
|  | p | 0.083 | | 0.072 | 0.441 | 0.089 | 0.032 | 0.889 | 0.549 | 0.997 | 0.077 | 0.809 | 0.04 | 0.119 |
| Thalamo-  occipital distance | r | -0.076 | | 0.137 | -0.225 | 0.137 | 0.058 | 0 | 0.095 | -0.064 | -0.215 | 0.065 | 0.087 | 0.049 |
|  | p | 0.299 | | 0.061 | 0.002 | 0.06 | 0.431 | 0.999 | 0.194 | 0.383 | 0.003 | 0.38 | 0.233 | 0.523 |
| Inter-hemispheric  distance | r | -0.497 | | 0.127 | -0.012 | 0.321 | 0.322 | 0.008 | 0.082 | 0.166 | -0.116 | 0.235 | 0.408 | 0.345 |
|  | p | <0.001 | | 0.084 | 0.865 | <0.001 | <0.001 | 0.915 | 0.261 | 0.023 | 0.113 | 0.001 | <0.001 | <0.001 |

Abbreviations: C/S, Caesarean section; IUGR, intrauterine growth restriction; PDA, patent ductus arteriosus; CLD, chronic lung disease; Low Apgar, Apgar scores <6 at 5 minutes after birth.

Delayed feeding, is defined as enteral feeding < 100ml/kg/day on Day 7.

P-values are from the Pearson’s or Spearman’s correlation coefficient.

**Online Supplemental Fig. 1: Relationships between regional brain sizes and age**


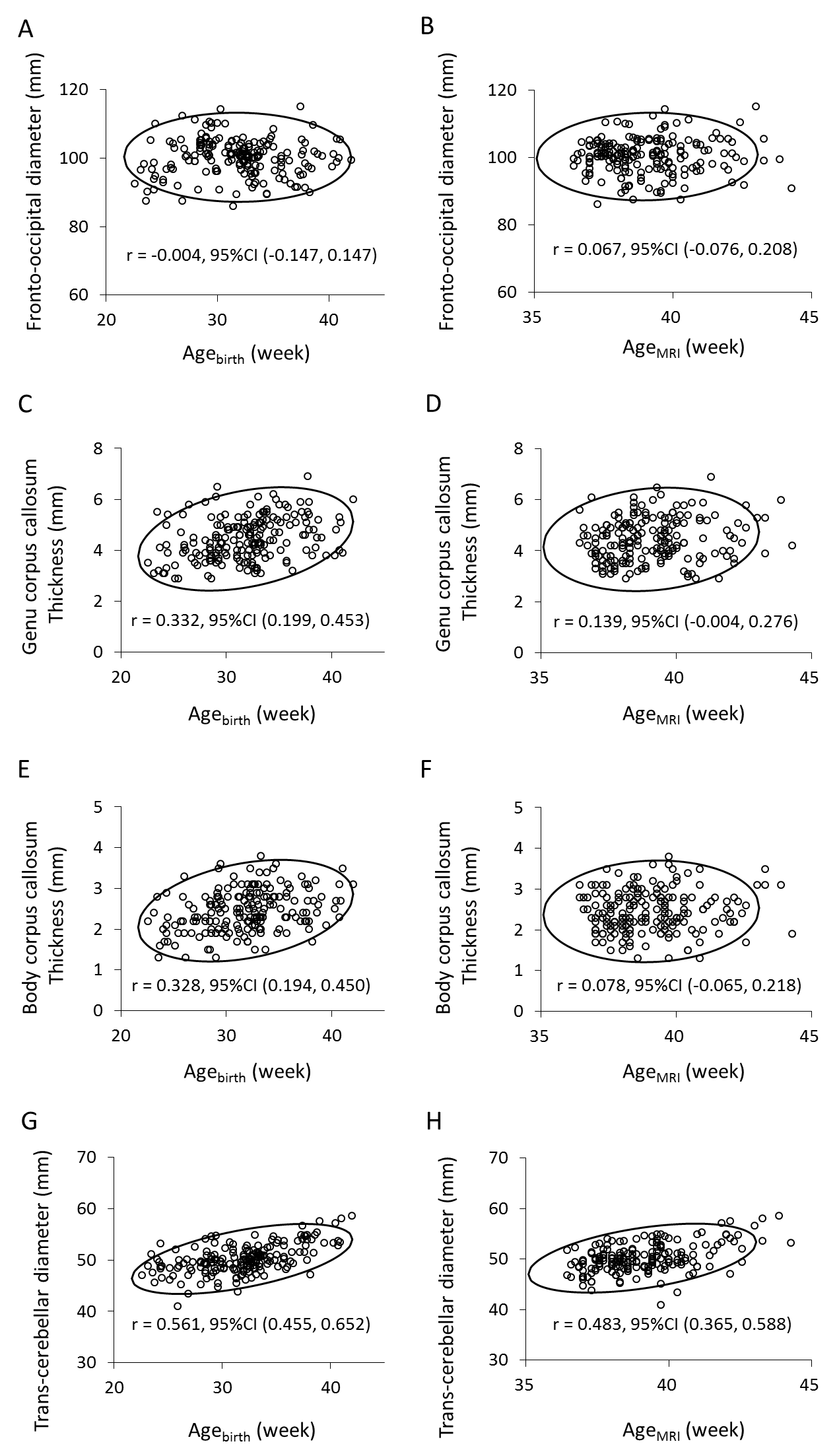


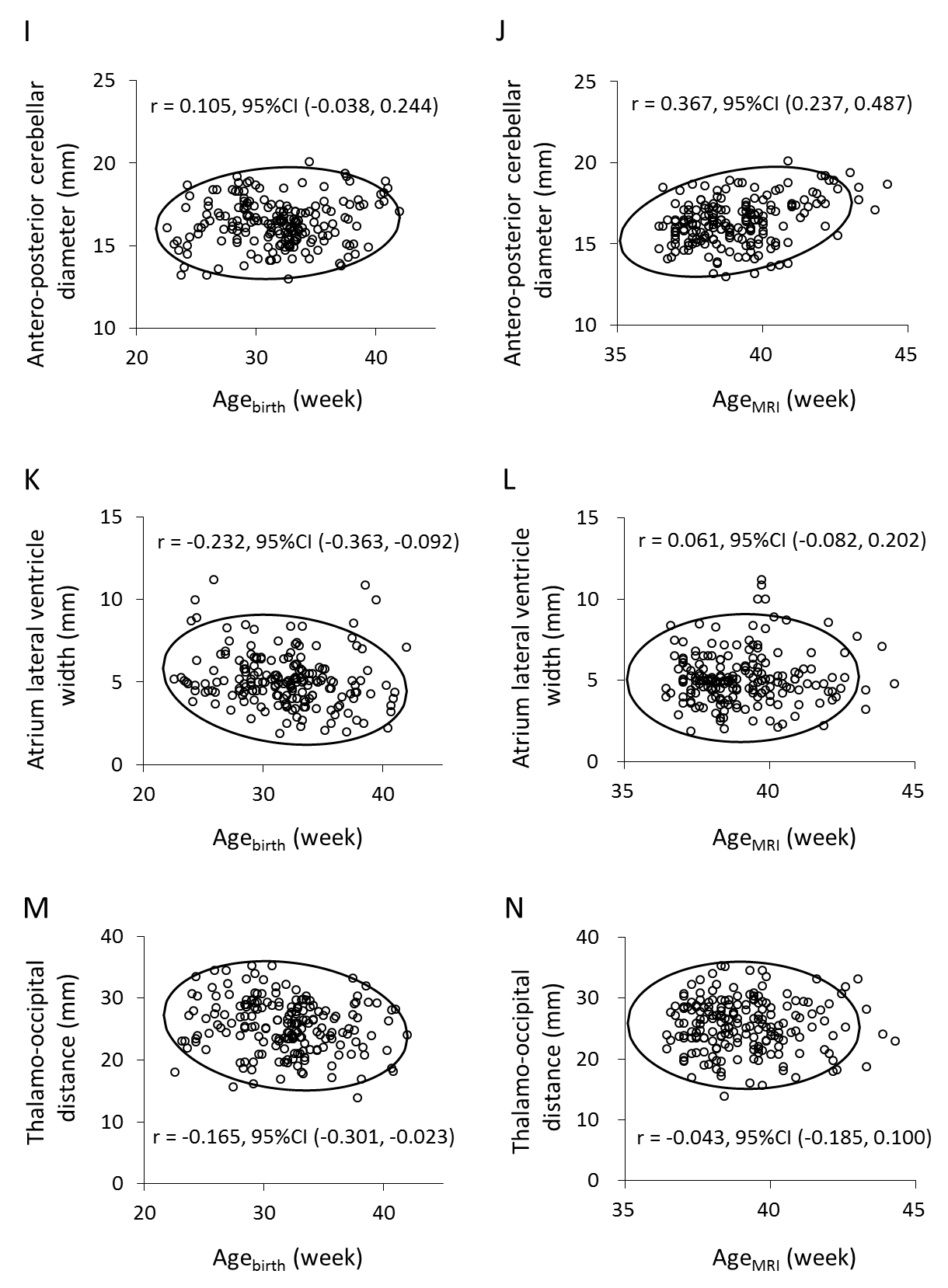


Regional brain sizes are plotted against Age_birth_ (A, C, E, G, I, K and M) and Age_MRI_ (B, D, F, H, J, L and N) with 95% confidence ellipse. The front-occipital diameter (A and B) was not correlated with Age_birth_ and Age_MRI_. The thickness of the corpus callosum (genu and body) (C, D, E and F), width of atrium lateral ventricle (K and L) and the thalamo-occipital distancewere (M and N) correlated with Age_birth_, but not Age_MRI_. The antero-posterior cerebellar diameter (J and I) was correlated with Age_MRI_, but not Age_birth_. The trans-cerebellar diameter (G and H) was positively correlated with both Age_birth_ and Age_MRI_.

Abbreviations: Age_birth_, gestational age at birth. Age_MRI_, corrected age at MRI scan. CI, confidence interval.

**Online Supplemental Fig. 2: Representative MRI of newborn infants in the study cohort**

**
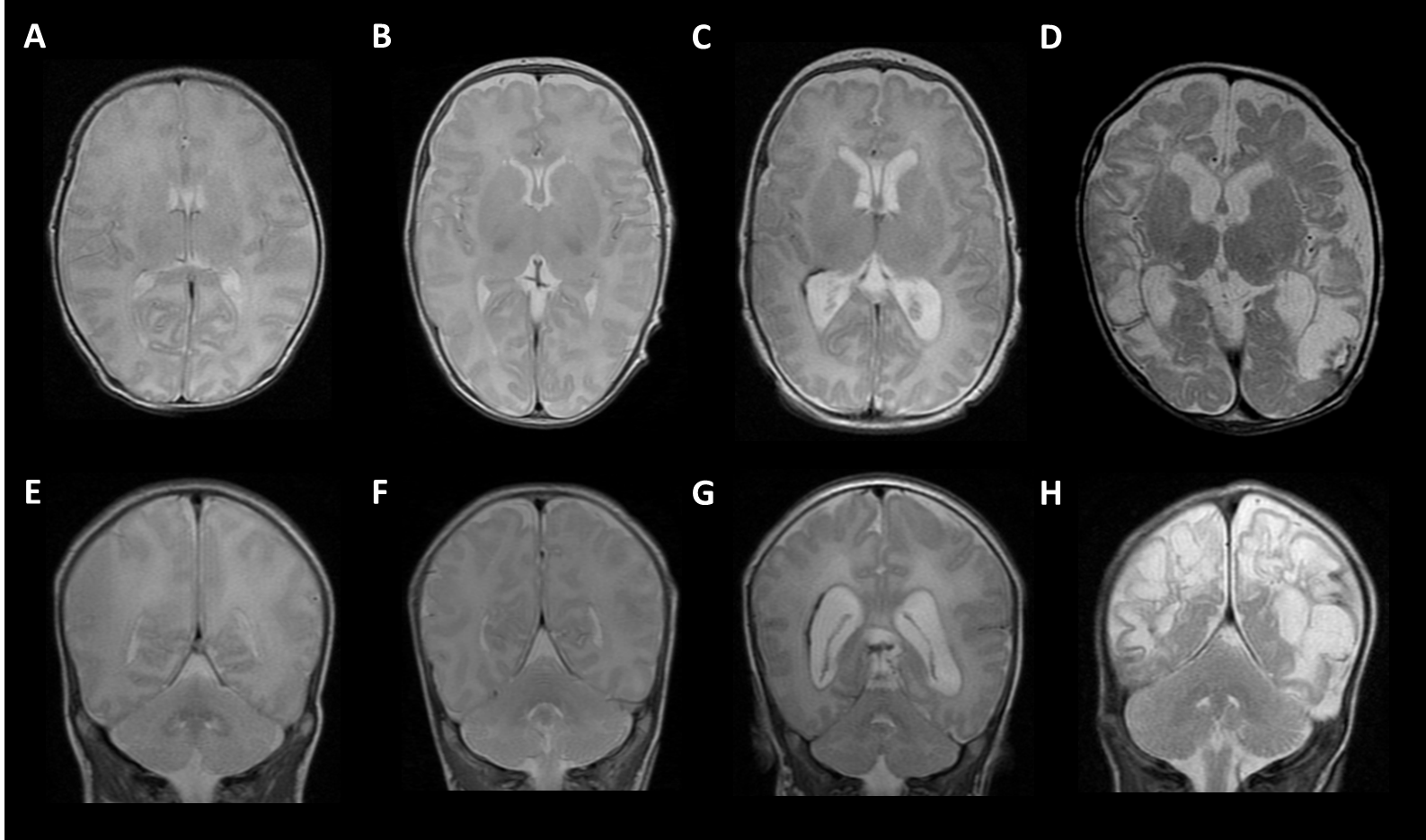
**

Representative axial (A-D) and coronal (E-H) T2-weighted MRI demonstrating none, mild, moderate and severe brain injury assessed using an established MRI scoring system for brain maturation, growth and injury (Kidokoro et al. Pediatrics 2014). Note that all newborn infants with severe brain injury simultaneously showed severe destructive brain injury of either intra-ventricular haemorrhage ≥ grade 3, cerebral venous or arterial infarction, or cystic encephalomalacia, and therefore, these infants were excluded from the further analysis.
